# COVID-19 and psychiatric disorders among young people: a cross-sectional study

**DOI:** 10.1097/YIC.0000000000000565

**Published:** 2024-08-22

**Authors:** Tiziano Prodi, Filippo Dragogna, Beatrice Benatti, Alberto Varinelli, Simone Vanzetto, Letizia Gianfelice, Bernardo Dell’Osso

**Affiliations:** a Department of Mental Health, Department of Biomedical and Clinical Sciences “Luigi Sacco”, University of Milan; b Department of Mental Health, Psychiatry 1 Unit, Fatebenefratelli Hospital, Milan; c Department of Mental Health, ASST Brianza, Vimercate; d “Aldo Ravelli” Center for Nanotechnology and Neurostimulation, University of Milan, Milan, Italy; e Department of Psychiatry and Behavioral Sciences, Stanford University, Stanford, California, USA; f Centro per lo studio dei meccanismi molecolari alla base delle patologie neuro-psico-geriatriche, University of Milan, Milan, Italy

**Keywords:** adolescent, anxiety, COVID-19, feeding and eating disorders, first psychiatric episode, sleep initiation and maintenance disorders, substance-related disorders

## Abstract

During the COVID-19 pandemic, a significant rise in mental health issues was observed. Particularly, children and adolescents have shown a higher risk of developing mental disorders than adults. This study aimed to describe the evolving features of the requests for psychiatric emergency interventions during the COVID-19 pandemic in young people. We conducted a cross-sectional study comparing the number, characteristics, and symptoms of people aged between 12 and 18 years old attending one Emergency Department (ED) for psychiatric issues, considering three different periods: T0 (8 March 2019–7 March 2020), T1 (8 March 2020–7 March 2021), and T2 (8 March 2021–7 March 2022). Total admissions were 220: 99 (45%) during T0, 40 (18.1%) for T1, and 81 (36.8%) for T2 (*P* < 0.001). A significant decrease in the mean age from T0 to T1 was found (*P* < 0.01). Admissions for psychomotor agitation decreased, while admission due to anxiety disorder and nonsuicidal self-injury raised significantly (*P* < 0.05), as for first psychiatric presentation (*P* < 0.01). Regarding substance use, a significant reduction was observed (*P* < 0.05). The rates of eating disorders (*P* < 0.001) and early insomnia (*P* < 0.01) increased from T0. These findings highlight the worsening of psychiatric symptoms in the young population during the COVID-19 pandemic.

## Introduction

Since the outbreak of COVID-19, higher rates of depression, anxiety, distress, and insomnia have been reported in different groups of the population (Wu *et al*., 2021). Indeed, despite the clinical presentation of COVID-19 being dominated by respiratory signs, the virus infection may have different neuropsychiatric consequences due to systemic inflammation, infection of neural cells by SARS-COV-2, neuroinflammation, glial dysfunction, or aberrant epigenetic modifications of stress-related genes (Steardo *et al*., 2020). In order to prevent disease transmission, during the early phase of the pandemic, different countries implemented quarantine measures like nationwide lockdown programs; these kinds of measures might produce mass anxiety and distress, due to factors like the sense of getting cornered and loss of control (Dubey *et al*., 2020). In Italy, a nationwide lockdown was imposed from 9 March to 3 May 2020; during that period, the movement of the population was restricted, except for work and health reasons: at the end of the lockdown period, on 4 May, the economic activities were gradually resumed, and the restrictions were partially eased (Capuzzi *et al*., 2022). In Lombardy, in order to respond to the COVID-19 outbreak, important changes occurred in the management of the Departments of Mental Health and Addiction (DSMD): some psychiatric wards were rearranged in order to admit COVID-19 patients and many outpatient clinics had to restrict appointments to most urgent patients only (de Girolamo *et al*., 2020).

Stressors like prolonged quarantine, fear of infection, frustration, boredom, inadequate supplies, inadequate information, financial loss, and stigma led to mental health issues in the general population (Hossain *et al*., 2020). Moreover, other factors such as job insecurity, adverse employment environment, work rights exploitation, and uncertainty of the future worsened preexisting psychological conditions, especially in younger people and those with a higher educational background (Giorgi *et al*., 2020). In fact, according to a recent review, in the first 2 months following the WHO declaration of the COVID-19 pandemic, a significant increase in mental health symptoms compared to the previous period was observed (Robinson *et al*., 2022) as well as an increase in distress levels and depressive and anxiety symptoms in the general population (Manchia *et al*., 2022). Conversely, some psychiatric conditions such as obsessive-compulsive disorder have progressively worsened throughout the pandemic period (Benatti *et al*., 2022).

More in detail, children and adolescents who experienced COVID-19 have a higher risk of developing mental disorders than adults (Liu *et al*., 2021); they are also more vulnerable to mental health problems than adults when facing the same challenges (Bai *et al*., 2022). Previous research showed that the pediatric population that has experienced enforced isolation or quarantine was five times more likely to require mental health service input and experienced higher levels of post-traumatic stress (Loades *et al*., 2020). Furthermore, in young people, perceived stress affects mood, eating behaviors, and sleep (de Zambotti *et al*., 2018). Stressors such as adverse life events are associated with a higher risk of anxiety disorders onset (Miloyan *et al*., 2018), particularly stressful life events in the last year may lead to anxiety disorder, post-traumatic stress disorder, or major depressive disorder onset (McLaughlin *et al*., 2010). In general, anxiety disorders represent the most common mental condition among children and adolescents and affect approximately 6.5% of this population (Polanczyk *et al*., 2015; Bai *et al*., 2022). Furthermore, considering anxiety symptoms in the pediatric population, sleep anxiety is often correlated with insomnia (Asarnow and Mirchandaney, 2021). While, regarding specifically adolescents, a link between stress and insomnia was reported (Blake *et al*., 2018); indeed, adolescents with insomnia had larger cortisol awakening responses compared to adolescents without sleep disturbances (Zhang *et al*., 2014; Blake *et al*., 2018). As reported in a recent review, during the COVID-19 pandemic an increase in mental health concerns and the prevalence of suicidal ideation, suicide, and nonsuicidal self‐injury among children and adolescents was described (Samji *et al*., 2022). According to diathesis-stress explanations, predisposing biological, personality, and cognitive vulnerabilities combined with exposure to negative life events – including both early and recent life adversity, and psychiatric disorders – may increase the risk of suicidal behaviors in adolescence (Evans *et al*., 2004). Furthermore, due to the elevated levels of impulsivity and emotional reactivity, adolescence is considered a generally vulnerable phase for developing nonsuicidal self-injury behavior (Brown and Plener, 2017).

After the implementation of lockdown measures a reduction in the Emergency Department (ED) referral rates for mental health concerns was reported, showing a new rise in psychiatric consultations in EDs after the lockdown conclusion (Chen *et al*., 2020; Carrasco *et al*., 2021). An interesting Italian multicentric study reported a 30% global reduction in the ED psychiatric consultations in 2020 compared to 2019: particularly, during the lockdown period the majority of patients who entered the ED already had a previous admission to psychiatric ward, if compared to the same period in 2019; conversely, at the end of lockdown we assisted to an increase in the admissions in the psychiatric wards, especially for patients with more severe conditions and with more recent onset of disorder, when compared to the same period in 2019 (Balestrieri *et al*., 2021). According to these results, the reduction of ED psychiatric consultations for children and young adults during the lockdown period may have led to delays in the appropriate support and treatment in the earlier stages of life, worsening the severity of mental health symptoms, and resulting in poorer health outcomes (Samji *et al*., 2022).

Hence, the present study aimed to describe the frequency, features, and symptoms of the young population attending the ED of a large metropolitan hospital located in Milan, Italy for psychiatric issues during the lockdown period, then compare the same period to 2019 and 2022. At the same time, we aimed to describe the evolving characteristics in the aid requests during the COVID-19 pandemic to evaluate the impact of pandemic in young people mental health.

## Material and methods

We conducted a cross-sectional observational study at the ASST Fatebenefratelli Sacco in Milan, Italy. We compared the frequency, sociodemographic features, and psychiatric symptoms of subjects aged between 12 and 18 years attending the ED of Fatebenefratelli Hospital for psychiatric issues in three different periods. We defined T0 as the period between 8 March 2019 and 7 March 2020, T1 as the period between 8 March 2020 and 7 March 2021, and T2 as the period between 8 March and 7 March 2022. Particularly, we collected data regarding mean age, gender, place of living, if adopted, educational status, if born in Italy, foreign origin, previous COVID-19 infection (if applicable), COVID-19 vaccination (if applicable), personal and family psychiatric history, alcohol or substance use, reasons for the emergency consultation, type of ED discharge, and psychiatric diagnosis according to ICD9. All the consecutive subjects aged between 12 and 18 years old referred to the psychiatric ED were included in the present study, there were no exclusion criteria.

Data were collected anonymously through the medical records by three residents in psychiatry, they were trained by an expert researcher to achieve consistency. After data collection, the integrity of the database was checked by comparing it with all the medical records by a different psychiatrist. Approval was granted by the Ethics Committee of Milan Area 1, n. 2023/ST/02. Informed consent for data collection for research purposes was obtained at the time of ED admission.

Since a nonparametrical distribution was found, Chi-squared test and Mann–Whitney U test for categorical variables and continuous variables, respectively, were used. Data were analyzed using SPSS v. 24 (IBM Corp., Armonk, New York, USA).

## Results

Considering the whole timeframe of the study, total admissions in ED for psychiatric issues in 12–18 years old subjects were 220. The mean age was 15.76 ± 0.43 years and 38.18% were males. Sociodemographic data were summarized in Table 1.

**Table 1:** Sociodemographic and clinical features

	Total *N* = 220 (%)	T0 *n* = 99 (45%)	T1 *n* = 40 (18.1%)	T2 *n* = 81 (36.8%)
Mean age (years)	15.76 ± 0.43	16.07 ± 1.63	15.05 ± 1.77	15.74 ± 1.47
Gender (F : M)	136 (61.82%) : 84 (38.18%)	58 (58.59%) : 41 (41.41%)	25 (62.5%) : 15 (37.5%)	53 (65.43%) : 28 (34.57%)
Place of living				
Biological family	164 (74.55%)	67 (67.68%)	33 (82.5%)	64 (79.01%)
Adoptive family	9 (4.09%)	7 (7.07%)	1 (2.5%)	1 (1.23%)
Psychiatric community	45 (20.45%)	25 (25.25%)	5 (12.5%)	15 (18.52%)
Relatives	2 (0.91%)	0	1 (2.5%)	1 (1.23%)
Adopted (Yes)	10 (4.55%)	6 (6.06%)	2 (5%)	2 (2.47%)
Educational status				
Middle grade school	23 (10.45%)	6 (6.06%)	10 (25%)	7 (8.64%)
High school	89 (40.45%)	35 (35.35%)	15 (37.50%)	39 (48.15%)
Not attending	6 (2.73%)	4 (4.04%)	0	2 (2.47%)
Not known	103 (46.82%)	54 (54.55%)	15 (37.50%)	33 (40.74%)
Born in Italy (Yes)	174 (79.09%)	77 (77.78%)	30 (75%)	67 (82.72%)
Foreign origin (Yes)	78 (35.45%)	39 (39.39%)	16 (40%)	23 (28.40%)
Previous Covid-19 infection				
Yes	1 (0.45%)	0	1 (2.5%)	0
No	120 (54.55%)	0	39 (97.5%)	81 (100%)
Not applicable	99 (45%)	99 (100%)		
Vaccinated for COVID-19				
Yes	18 (8.18%)	0	0	18 (22.22%)
No	78 (35.46%)	0	15 (37.5%)	63 (77.78%)
Not applicable	124 (56.36%)	99 (100%)	25 (62.5%)	
First psychiatric episode	36 (16.36%)	7 (7.07%)	10 (25%)	19 (23.45%)
Reason for ED admission*				
Psychomotor agitation		46 (46.47%)	12 (30%)	25 (30.86%)
Nonsuicidal self-injury		11 (11.11%)	6 (15%)	21 (25.93%)
Anxiety/panic		16 (16.16%)	14 (35%)	18 (22.22%)
Psychosis		2 (2.02%)	0	0
Substance intoxication		16 (16.16%)	5 (12.50%)	7 (8.64%)
Depressive symptoms		4 (4.04%)	1 (2.50%)	1 (1.24%)
Other		4 (4.04%)	2 (5%)	9 (11.11%)
Previous contact with mental health services§				
Not known		41 (41.42%)	16 (40%)	25 (30.87%)
Psychotherapy		12 (12.12%)	3 (7.5%)	7 (8.64%)
Child neuropsychiatry outpatients’ clinic		31 (31.31%)	12 (30%)	36 (44.44%)
Private practice		15 (15.15%)	9 (22.5%)	13 (16.05%)
Substance use#				
Tetrahydrocannabinol		11 (11.11%)	5 (12.5%)	11 (13.58%)
Cocaine		3 (3.03%)	0	0
Multidrug users		14 (14.14%)	2 (5%)	1 (1.23%)
Alcohol use†		13 (13.13%)	6 (15%)	15 (18.52%)

Values for categorical and continuous variables are expressed in percentages and mean ± SD, respectively.

* χ² (Chi-square) = 23.32 *P*< 0.05.

§ χ² (Chi-square) = 5.847 *P* = 0.441.

# χ² (Chi-square) = 15.085 *P*< 0.05.

† χ² (Chi-square) = 0.997 *P* = 0.607.

Regarding admissions, 45% (*N* = 99) of the admissions were registered during T0, 18.2% (*N* = 40) during T1, and 36.8% (*N* = 81) in T2 (*P* < 0.001). The mean age during T0 was 16.07 ± 1.63 years, 15.05 ± 1.77 years in T1, and 15.74 ± 1.47 years in T2. T0 and T1 years mean age were significantly different (*P* < 0.01).

ED admissions for psychomotor agitation decreased from 46.46% during T0 to 30% during T1 and T2. Conversely, admissions due to anxiety/panic disorder (T0: 16.16%, T1: 35%, T2: 22.22%) and nonsuicidal self-injury (T0: 11.11%, T1: 15%, T2: 25.92%) increased (*P* < 0.05). No significant differences in admissions rates were found for both patients already attending mental health services and for patients who were already prescribed psychiatric treatments (Table 1).

Regarding first psychiatric episodes, data showed a significant difference comparing the three periods: during T0 first episodes were 7.07%, in T1 25%, and in T2 23.45% (*P* < 0.01).

**Fig. 1:**
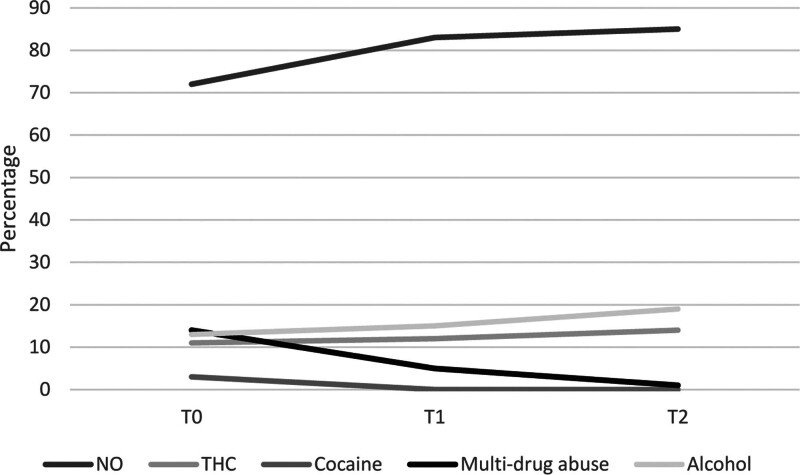
Substance and alcohol use rates over the three periods.

Significant differences were found in substance misuse during the three periods: patients who did not use substances increased (T0: 71.72%, T1: 82.5%, T2: 85.19%) and, at the same time, multidrug users decreased over the three periods (T0: 14.14%, T1: 5%, T2: 1.23%) (*P* < 0.05; Fig. 1). No significant differences were found regarding alcohol misuse (Table 1).

**Fig. 2:**
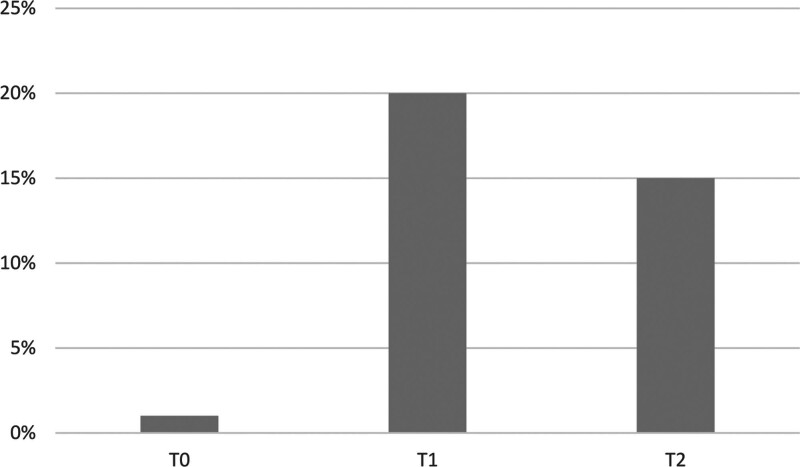
Eating disorders comorbidity rates among the three periods.

Regarding psychiatric comorbidities concomitant to the index episode, rates of eating disorders as comorbidities were 1.01% in T0, 20% in T1, and 14.81% in T2 (*χ*² = 16.021 *P* < 0.001; Fig. 2). Differences in other psychiatric comorbidities were NS.

**Fig. 3:**
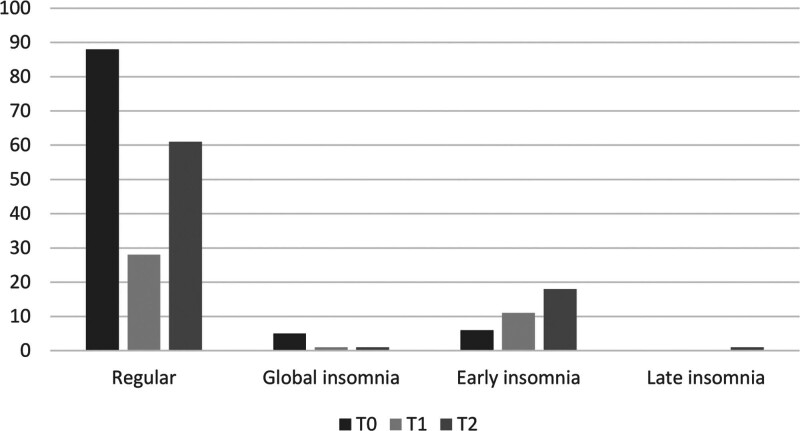
Sleep features across the three periods.

Considering sleep quality and sleeping disorders, we found a significant difference in subjects with regular sleep during the three periods: in T0 88.88% of the patients had regular sleep, 70% in T1, and 75.30% in T2. Consistently, early insomnia increased from 6.06% during T0 to 27.5% in T1; in T2 the percentage of subjects with early insomnia was 22.22%. Differences in regular sleep and early insomnia rates among the three periods were statistically significant (*χ*² =16.945 *P* < 0.01), no significant differences were found in other types of insomnia (Fig. 3).

No significant differences were found in terms of suicidality during the three periods; however, we found significant differences among the methods of nonsuicidal self-injury: the rates of drug poisoning were 46.15% in T0, 13.33% in T1, and 9.67% in T2, while the rates of jumping (T0: 0, T1: 6.66%, T2: 12.90%) and cutting (T0: 11.53%, T1: 33.33%, T2: 22.58%) as suicide attempts significantly increased from T0 to T1 and T2 (*P* < 0.01; Table 2).

**Table 2:** Suicidality

	T0 *n* = 99 (45%)	T1 *n* = 40 (18.1%)	T2 *n* = 81 (36.8%)
Suicide attempts* (Yes)	26 (26.26%)	15 (37.5%)	31 (38.27%)
Suicide attemps or self-injury methods§			
Nonsuicidal self-injury intentionality	11 (42.31%)	7 (46.67%)	17 (54.84%)
Drug poisoning	12 (46.15%)	2 (13.33%)	3 (9.68%)
Cutting	3 (11.54%)	5 (33.33%)	7 (22.58%)
Jumping	0	1 (6.67%)	4 (12.90%)

Values for categorical are expressed in percentages.

* χ² (Chi-square) = 3.424 *P* = 0.181.

§ χ² (Chi-square) = 13.858 *P* < 0.01.

The home discharge rates increased among the three periods (T0: 46.57%, T1: 62.5%, T2: 62.69%), as well as the admissions to the pediatric ward (T0: 8.57%, T1: 12.5%, T2: 17.28%); however, the admissions to the pediatric neuropsychiatric ward increased from 4.76% in T0 to 10% in T1; while, in T2 no admissions to the pediatric neuropsychiatric ward were registered (*P* < 0.01). The percentage of admissions to other mental health facilities (i.e. community centers) after discharge was 15.23% in T0, 5% in T1, and 7.40% in T2 (*P* < 0.01; Table 3). No differences were found in terms of discharge diagnoses.

**Table 3:** Discharge

	T0 *n* = 99 (45%)	T1 *n* = 40 (18.1%)	T2 *n* = 81 (36.8%)
Type of hospital discharge*			
Home	50 (50.51%)	25 (62.5%)	51 (62.96%)
Other mental health facility	14 (14.14%)	2 (5%)	6 (7.41%)
Admission in pediatric ward	6 (6.06%)	5 (12.5%)	14 (17.28%)
Admission in psychiatric ward	13 (13.13%)	3 (7.5%)	9 (11.11%)
Admission in neuropsychiatric pediatric ward	5 (5.05%)	4 (10%)	0
Left the ED before the discharge	6 (6.06%)	1 (2.5%)	1 (1.24%)
Moved to another ED	5 (5.05%)	0	0
Most relevant discharge diagnoses§			
Substance intoxication	15 (15.16%)	3 (7.5%)	7 (8.64%)
Anxiety disorder	22 (22.22%)	18 (45%)	23 (28.40%)
Eating disorder	2 (2.02%)	2(5%)	6 (7.41%)
Adjustment disorder	3 (3.03%)	0	2 (2.47%)
Personality disorder	12 (12.12%)	6 (15%)	12 (14.81%)
Post-traumatic stress disorder	3 (3.03%)	0	3 (3.70%)
Psychosis	1 (1.01%)	0	3 (3.70%)
Depression	7 (7.07%)	4 (10%)	5 (6.17%)
Conduct disorder	34 (34.34%)	7 (17.50%)	20 (24.70%)

Values for categorical are expressed in percentages.

* χ² (Chi-square) = 26.568 *P* = 0.009.

§ χ² (Chi-square) = 19.775 *P* = 0.231.

## Discussion

In the present study, we compared the clinical and sociodemographic variables of patients evaluated for psychiatric symptoms at the ED of Fatebenefratelli Hospital, ASST Fatebenefratelli Sacco in Milan, Italy in three different periods, from March 2019 to March 2022. The primary aim was to analyze whether and how the SARS-CoV-2 pandemic had a short-term influence on the access to emergency psychiatric services in Milan, one of the most affected cities by the pandemic in Italy.

The results showed significant differences among the three periods examined the pre-Covid period and the pandemic years.

We found a significant reduction in ED total admissions in T1 compared to T0 and a new rise in T2. This major decrease may be related to the specific context of the first 2 months of the pandemic, where social measures like lockdowns were established: in fact, several studies reported that between March and July of 2020 the number of EDs evaluated patients was significantly lower than the same months in 2019, while an increase in accesses has been reported from August 2020 and then remained stable, in 2021, and in the early months of 2022 (Garrafa *et al*., 2020; Bellan *et al*., 2021). According to Balestrieri *et al*. this finding might depend on many factors, including, but not limiting to, the fear of contagion and the difficulties in accessing to health services during the emergency (Balestrieri *et al*., 2021). At the same time, our data showed that children and adolescents admitted during T1 and T2 were younger than the ones admitted in the previous period: the reduction of the mean age might be due to stressors like home confinement, boredom, or lack of face-to-face contact with peers (Dubey *et al*., 2020). Conversely to this evidence, another study conducted in Italy found an increase in the mean age during the first 8 weeks after the COVID-19 outbreak (Davico *et al*., 2021). We, however, did not find a correlation between age reduction and specific diagnoses. This could be due to both the small sample size and the ED setting: on one side a bigger sample size (or better a multicentric design) could have helped to highlight also small differences increasing statistical power; on the other side, the ED setting could represent a limitation, indeed, some diagnoses such as eating disorders could refer to other clinical settings due to the nonurgent characteristics.

Another important finding of this study was the proportion of psychiatric disorder onset increased during T1 and T2 (*P* < 0.01), when COVID-19 pandemic social restrictions prevented social life. This data gain importance since loneliness – especially in adolescents – represents a risk factor for psychiatric disorders onset (Murata *et al*., 2021). According to our findings, the psychiatric onset increase in our ED might also be related to delayed psychiatric evaluation in outpatient facilities. Indeed, according to both national and regional rules implemented to prevent COVID-19 spread, psychiatric consultations in outpatient clinics were limited to patients with more urgent condition (de Girolamo *et al*., 2020). It is also reported that, during the lockdown period, people tried to avoid hospitals and clinics to obey the home confinement and fearing of getting infected (Clerici *et al*., 2020): this behavior may have contributed to a delayed presentation for clinical assessment, leading to the worsening of patients’ conditions and the consequent increase in ED admissions.

According to a systematic review, consumption of alcohol and other psychotropic substances among the general population increased during the pandemic (Roberts *et al*., 2021); conversely, considering the young population, isolation due to the pandemic was associated with a reduction of alcohol or other substance misuse (Bourduge *et al*., 2022; Temple *et al*., 2022). Consistently with that, a reduction of cocaine and multidrug consumption among adolescents across the whole timeframe of the study was observed; moreover, the proportion of people who did not use any substances increased in T1 and T2. Our findings may be partially explained by the reduction of social contact with peers due to lockdown measures: considering that adolescents generally use substances with peers, the reduction of social contact and the increase in time spent at home with family members may be responsible for the substance use decrease registered in our study (Bourduge *et al*., 2022). Hence, it is possible to assume that during the COVID-19 pandemic, a reduction in recreational use was registered. Our results, however, showed a nonsignificant rise in the percentage of cannabis and alcohol misuse. Further studies are needed to completely understand the relation between the COVID-19 pandemic and alcohol/substance misuse: in fact, an increase in cocaine and cannabis misuse among the general population and a rise in alcohol consumption rates among adolescents are both reported (Grigoletto *et al*., 2020; Capuzzi *et al*., 2022).

As reported in a recent systematic review, an association between the COVID-19 pandemic and higher rates of anxiety among adolescents has been confirmed by many studies (Jones *et al*., 2021). According to this evidence, an increase in the percentage of ED admissions due to anxiety or panic symptoms in T1 and T2, when compared to T0 (*P* < 0.05) was shown in the present paper. We hypothesize that social isolation and the lack of face-to-face relations are the possible grounds of the higher anxiety issues. Moreover, those two features are also risk factors for both anxiety and suicidal ideation (Farooq *et al*., 2021). Regarding suicidal behavior, our data showed a significant increase in the percentage of ED admissions due to nonsuicidal self-injury in T1 and T2 when compared to T0 (*P* < 0.05). However, despite no significant differences in suicidality, nonsuicidal self-injury is considered a predictor of future suicidal attempts in young people (Clarke *et al*., 2019). Particularly, nonsuicidal self-injury is often preceded by suicide ideation, and it may reduce inhibition through habituation to self-injury (Whitlock *et al*., 2013; Glenn *et al*., 2017). It is possible that the increase in anxiety symptoms discussed above led to higher rates of nonsuicidal self-injuries: the positive association between high levels of anxiety and depressive symptoms and the incidence of nonsuicidal self-injury among adolescents is largely reported in literature (Fergusson *et al*., 2000; Evans *et al*., 2004; Hawton *et al*., 2012; Moran *et al*., 2012). Furthermore, the self-harming behavior phenomenon may have been underestimated, since, according to Hawton and colleagues, presentation to a hospital occurs in only about one in eight adolescents who self-harm in the community (Hawton *et al*., 2012).

Regarding comorbidities, we found a significant increase in the percentage of eating disorders in T1 and T2 when compared to the previous period (*P* < 0.001). Our findings are in accordance with the literature: in a recent systematic review, an increase in the number of hospital admissions due to eating disorders during the COVID-19 pandemic compared to the previous year was reported (Devoe *et al*., 2023). Moreover, the worsening of eating disorders’ symptoms in the pediatric population during the lockdown period has been largely reported (Baenas *et al*., 2020; Monteleone *et al*., 2021; Nisticò *et al*., 2021). It is possible that the worsening of eating disorders’ symptoms was caused by the reduced access to mental health services and by the reduction of in-person treatments. However, according to Nisticò *et al*., patients with eating disorders had more chance to experience higher levels of stress, anxiety, and depressive symptoms than healthy subjects (Nisticò *et al*., 2021). Conversely, as highlighted in an Australian study, COVID-19 restrictions may have triggered the onset of eating disorders rather than worsened symptoms in subjects with preexisting eating disorders (Springall *et al*., 2022). The increase of eating disorders comorbidity and its worsening represents an important finding, due to the higher mortality rates associated (Smink *et al*., 2012).

Regarding sleep disorders, early insomnia percentage raised after the COVID-19 outbreak (*P* < 0.01). This evidence was in line with the general worsening of mental health conditions among adolescents, leading to the assumption that dysfunctional coping strategies and the novelty stressor affected sleep quality and particularly early insomnia, as supported also by our findings (Morin *et al*., 2021).

The present study had some limitations. First, data were collected in an ED setting with a brief evaluation and data may not always be accurate: the psychiatric diagnoses of mental disorders were made by different psychiatrists without psychometric evaluation, due to the emergency setting; moreover, some of the collected data were self-reported by patients. Moreover, also the reasons for the emergency consultation could reflect the habits of the triage service, rather than an actual diagnostic trend. Second, our study was conducted in a hospital located in the metropolitan city center: our findings might not be extended to people living in different contexts such as smaller cities. Third, the study was conducted in Lombardy, one of the most affected regions by the pandemic in the world. Fourth, we didn’t consider the lockdown and the post-lockdown periods precisely: as mentioned in the introduction, the first lockdown period in Italy was from 9 March to 3 May 2020. Fifth, according to the medical records of our hospital, we made diagnoses according to ICD9: using ICD9 instead of the more recent ICD11 may have caused a reduction in the transferability of our findings. Last, the small sample size – especially in the T1 period – could also be considered a limitation in the generalizability of our results.

Future perspectives include the follow-up of young patients after ED discharge to assess the appropriateness of the emergency presentations.

Despite some limitations, this study showed a worsening of psychiatric symptoms in the young population that might be more affected by the social consequences of the COVID-19 pandemic. Even if our data showed a decrease in the admissions to ED for psychiatric issues and a reduction in substance misuse, they also enlighten a rise in psychiatric disorders onsets, a worsening of sleep quality, and an increase of eating disorders among young people during COVID-19 pandemic. Furthermore, the reduction of the mean age of subjects admitted to ED for psychiatric reasons might reflect a weakening of mental health among younger people in general; however, further studies are needed to evaluate the correlation between the COVID-19 pandemic and the worsening of mental health in young patients.

## Acknowledgement

The present study was supported in part by Fondazione Cariplo, grant 2021-4490.

T.P. contributed to conceptualization, data curation, investigation, writing the original draft, reviewing, and editing; F.D. contributed to conceptualization, data curation, reviewing, and supervision; B.B. contributed to reviewing and supervision; A.V. contributed to formal analysis and reviewing; S.V. contributed to conceptualization, data curation, and writing the original draft; L.G. contributed to investigation, data curation, and writing the original draft; B.D. contributed to conceptualization, reviewing, and supervision.

### Conflicts of interest

There are no conflicts of interest.
